# Role of Damage-Associated Molecular Pattern/Cell Death Pathways in Vaccine-Induced Immunity

**DOI:** 10.3390/v13122340

**Published:** 2021-11-23

**Authors:** Sun Min Lee, Paul Kim, Jinsuh You, Eui Ho Kim

**Affiliations:** Viral Immunology Laboratory, Institut Pasteur Korea, Seongnam-si 13488, Korea; sunmin.lee@ip-korea.org (S.M.L.); paul.kim@ip-korea.org (P.K.); jinsuhyou@naver.com (J.Y.)

**Keywords:** damage-associated molecular pattern, cell death, vaccine, immunity

## Abstract

Immune responses induced by natural infection and vaccination are known to be initiated by the recognition of microbial patterns by cognate receptors, since microbes and most vaccine components contain pathogen-associated molecular patterns. Recent discoveries on the roles of damage-associated molecular patterns (DAMPs) and cell death in immunogenicity have improved our understanding of the mechanism underlying vaccine-induced immunity. DAMPs are usually immunologically inert, but can transform into alarming signals to activate the resting immune system in response to pathogenic infection, cellular stress and death, or tissue damage. The activation of DAMPs and cell death pathways can trigger local inflammation, occasionally mediating adaptive immunity, including antibody- and cell-mediated immune responses. Emerging evidence indicates that the components of vaccines and adjuvants induce immunogenicity via the stimulation of DAMP/cell death pathways. Furthermore, strategies for targeting this pathway to enhance immunogenicity are being investigated actively. In this review, we describe various DAMPs and focus on the roles of DAMP/cell death pathways in the context of vaccines for infectious diseases and cancer.

## 1. Introduction

Through the course of history, fatal viral or bacterial infections have posed a threat to human health, and vaccines against these pathogens have been continuously developed. For example, during the Spanish flu, the first pandemic caused by the influenza virus, more than 20 million cases of mortality were reported among 500 million infected individuals from 1918 to 1920. After the first isolation of human influenza virus in 1933 [1], a live attenuated influenza vaccine (LAIV) adapted to rodents, which would cause mild symptoms in humans, was developed. However, LAIV, which was almost identical to the live virus, exhibited safety and stability issues, thereby necessitating the introduction of an inactivated influenza vaccine (IIV). Influenza virus inactivated by high temperatures or chemical agents such as formaldehyde can effectively induce antibody production [2]. In the 1960s, split and subunit vaccines, which were new inactivated formulations, were tested in children under more stringent safety criteria [3]. These types of vaccines primarily contain the viral surface antigens such as hemagglutinin and neuraminidase instead of whole viruses, as the former can induce antibody responses. Although the action of these vaccines was actively investigated in the 1970s and 1980s, they were less immunogenic than LAIV and the previous version of IIV. It has been acknowledged that attempts to reduce adverse reactions and risks may have also reduced the efficacy of different vaccines [4]. Based on these findings, in the 1990s an adjuvant was used in FLUAD, an inactivated influenza vaccine for the elderly in order to enhance vaccine efficacy. In fact, various adjuvants have been applied to vaccines, commencing with the use of aluminum-based adjuvants in the 1930s, to modulate the efficacy and safety of vaccines. Aluminum salts, the most commonly used adjuvants, have been widely used for decades in vaccines against diphtheria, tetanus toxoids, pertussis, hepatitis, human papillomavirus, poliovirus, and influenza. Since the 2000s, the range of adjuvants has expanded to oil-in-water emulsions, monophosphoryl lipid A (MPLA), CpG DNA, and saponin-based adjuvants with approval from the Food and Drug Administration (FDA) of USA [5].

Although adjuvants have been commonly used to increase vaccine efficacy, the mechanisms underlying their actions are not well defined. As a means to complement vaccine efficacy and safety, tremendous efforts have been made to understand the roles of pathogen-associated molecular pattern (PAMP) as well as the mechanism underlying vaccine antigenicity [6]. For example, many researchers have shown that innate immunity can be induced through PAMPs via immunization with live attenuated vaccines, such as YF-17D and BCG, with the vaccines against yellow fever and tuberculosis being the most effective [7]. Moreover, several recent studies have reported that squalene emulsion-based vaccine adjuvants such as AddaVax and MF59 stimulate damage-associated molecular patterns (DAMPs) or cell death signaling pathways [8]. It has been proposed that various types of adjuvants, including alum and MF59, are associated with DAMPs and cell death pathways to mount optimal immunogenic responses [7,9].

Host cells possess sensors to distinguish non-self (exogenous) from self (endogenous) molecules, which are operated by pattern recognition receptors (PRRs), including Toll-like receptors (TLRs), NOD-like receptors (NLRs), RIG-I-like receptors, and C-type lectin receptors. It is well known that PRRs recognize molecules derived from pathogens (pathogen-associated molecular patterns, PAMPs) to initiate immune responses [10]. Typical PAMPs include microbial components such as lipopolysaccharide (LPS), peptidoglycan, lipoproteins, and microbial genomic fragments (RNA/DNA) [10].

Conversely, DAMPs, triggered by cellular stress and death or tissue injury, comprise a wide range of molecules derived from the extracellular matrix (e.g., proteoglycans) and those found in normal cells (e.g., DNA, RNA, uric acid, ATP, HMGB1, histones, and IL-1α) [11]. Once released, DAMPs can mediate inflammatory responses, antitumor immunity, and tissue repair [12]. Particularly, with respect to vaccine-induced immunity, certain vaccine adjuvants are known to induce DAMPs and initiate or intensify immunogenicity. These adjuvants, referred to as DAMP adjuvants, include alum, oil-in-emulsion adjuvants, 2-hydroxypropyl-β-cyclodextrin (HP-β-CD), and saponin-based adjuvants.

This review focuses on the role of DAMPs/cell death in vaccine-induced immunity. We will first describe the mechanisms underlying generation and action of various DAMPs that are associated with vaccines, and discuss the function of DAMPs in the immunity induced by vaccines and adjuvants. Finally, we will discuss the potential applications of DAMPs and cell death pathways as targets for the development of effective vaccines.

## 2. Mechanisms Underlying DAMP Release and Actions

DAMPs are molecules generated either through active secretion from live cells or through passive release following cell death, such as apoptosis, and accidental or regulated necrosis, such as pyroptosis, necroptosis, and ferroptosis [13]. The types of DAMPs differ depending on the cell death type and stage, and each DAMP has been shown to perform distinct functions and exhibit different modes of action.

### 2.1. DNA

DNA is composed of nucleotides (structured with a phosphate group, a deoxyribose sugar, and a single nitrogen-containing base) and carries the genetic code of organisms in the cell nucleus, governing biological functions. In addition to its basic function as a source of genetic information, when it is present outside cells, nuclear or mitochondrial DNA can stimulate the immune system via diverse cell death pathways or exosome/ectosome release [13,14,15]. Nucleus-derived extracellular DNA is generally inactive due to the fact that the DNA sensors in cells primarily recognize CpG motifs that are suppressed in mammalian nuclear DNA but expressed in bacterial DNA [16]. However, extracellular DNA can acquire immunostimulatory properties by forming complexes with other DNA-binding proteins [15]. For example, HMGB1 enables DNA to enter cells to interact with the endosomal DNA sensor TLR9. In contrast, owing to the high content of unmethylated CpG motifs, mitochondrial DNA exhibits activity independently, stimulating PRRs [17]. The majority of DNA sensing occurs through TLR9, absent in melanoma 2 (AIM2), interferon-inducible protein 16 (IFI16), cyclic GMP-AMP synthase (cGAS), and stimulator of interferon genes (STING). When these receptors recognize perturbations in extracellular DNA homeostasis, they trigger innate immune responses. TLR9 signaling stimulates NF-κB and interferon regulatory factor (IRF) pathways [18,19]. DNA recognition by AIM2 or IFI16 activates inflammasomes to induce the production of pro-inflammatory cytokines (IL-1β and IL-18) [20,21]. In addition, the cytosolic DNA sensing pathway mediated by cGAS-STING induces the activation of the NF-κB pathway, leading to IL-6 and tumor necrosis factor (TNF) production [22]. The activation of NF-κB pathway also mediate the anti-apoptotic signaling in DCs by inhibiting expression of a pro-apoptotic gene, Bim [23].

### 2.2. Uric Acid

Uric acid is the final product of purine metabolism. Owing to its pro-inflammatory properties, uric acid has been implicated in kidney disease [24], Alzheimer’s disease [25], and gout [26,27]. Several recent studies have demonstrated the characteristics of uric acid as a DAMP [28]. Uric acid can be generated during massive cell damage or cell death as well as from the breakdown of exogenous purines. While uric acid is soluble inside cells, excess uric acid in serum may undergo saturation and form monosodium urate (MSU) crystals in the extracellular matrix. In humans, only a high concentration of the crystallized form of uric acid exhibits pro-inflammatory properties; the soluble form (sUA) shows no significant pro- or anti-inflammatory activity [29]. This phenomenon is consistent with the phenomena reported in rhesus monkeys or great apes [30,31]. In rodents, both forms stimulate pro-inflammatory responses [31]. Braga et al. suggested that the species-dependent difference may be due to the evolutionary deletion of the Naip1-Nlrp3 inflammasome platform, which is involved in the murine recognition of sUA. 

With respect to the mechanism underlying inflammatory effects mediated by uric acid crystals, it is known that uric acid crystals activate NLRP3 inflammasomes with the consequent release of IL-1β and IL-18, which may induce neutrophil influx [27]. Kono et al. demonstrated that uric acid derived from sterile cell death contributes to inflammatory responses in mice [28]. Uric acid is known to directly influence various immune cells, including macrophages and T and B lymphocytes, as shown in different studies on human peripheral blood mononuclear cells. Uric acid stimulates the pro-inflammatory activity of in vitro cultured monocyte-derived macrophages by increasing the production of inflammatory factors (TNF-α, TLR4, and CD11c) and promoting macrophage phagocytosis [32]. In addition, in isolated human T cells, urate crystals elevated the zeta chain phosphorylation of the T-cell receptor (TCR) complex, indicating the direct activation of the TCR complex [33]. Similarly, urate crystals trigger B cell receptor signal transduction and induce B cell proliferation in vitro [34]. Recently, it has been shown that high levels of uric acid increased IL-1β and IL-1Ra production in human peripheral blood mononuclear cells freshly isolated from healthy people, as examined in vitro [35]. Furthermore, whole blood DNA from hyperuricemic people had different methylation features in genes related to inflammatory cytokine signaling as compared to those from normouricemic people [35]. 

### 2.3. Adenosine Triphosphate (ATP)

ATP, a nucleoside triphosphate, functions as the primary molecule for energy metabolism by hydrolyzing into ADP and phosphate in cells. However, when ATP molecules are present outside cells, they act as signaling molecules [36]. Typically, ATP is generated in the nervous system as a co-transmitter with neurotransmitters and plays particular roles in specific pathways associated with the five senses (sight, smell, taste, touch/pain, and sound), breathing, and cardiac and gastrointestinal functions. Extracellular ATP is present in the healthy tissues at negligible levels (nanomolar levels), but the level increases considerably in an inflammatory microenvironment [37,38]. Under inflammatory conditions, pro-inflammatory stimuli, such as LPS, reactive oxygen species (ROS), and hypoxia lead to extracellular ATP generation. The chemical properties of ATP, including low molecular weight and negative charge, facilitate translocation [39]. The major release mechanisms include necrosis, apoptosis, and pyroptosis, and the release channels include lysosomes, exosomes, and channel pores [13]. Once generated, ATP activates purinergic P2X/P2Y receptor signaling, which affects the host defense against infectious microbes or the outcomes of inflammatory/infectious disorders [40]. For example, P2Y_2_R signaling mediates the clearance of mucus during inflammatory diseases and dermal wound healing [41]. In terms of cell death, extracellular ATP generated by apoptotic cells acts as a “find-me” signal via P2Y_2_R to prompt the clearance of apoptotic cells [42]. In addition, ATP-P2Y_2_R signaling contributes to migration and cytokine/DAMP production by neutrophils [43] or eosinophils [44]. The P2Y_6_R pathway is also important in innate immunity owing to its ability to trigger phagocytosis. However, it can induce chronic inflammatory diseases, such as inflammatory bowel diseases [45] and asthma [46]. P2Y_12_R signaling affects lung function in asthma in humans through the activation of dendritic cells (DCs) that promote allergic T cell activity [47]. ATP-induced P2X_7_R signaling contributes to the host defense mechanism against pathogenic infection by promoting inflammasome activation in macrophages [48]. However, the stimulation of DCs by ATP-P2X_7_R signaling primes CD8^+^ T cells, CD4^+^ T cells, or Th17 cells to promote chronic inflammation during dermatitis, asthma, psoriasis, and inflammatory bowel disease [42]. Under the inflammatory conditions, Th1 cells can also differentiate into Th17 cells which may be pathogenic in inflammatory bowel disease [49].

### 2.4. High Mobility Group Box Protein 1 (HMGB1)

The high mobility group (HMG) is a group of nuclear proteins first isolated and characterized in 1973 [50,51] and named based on their high mobility in gel electrophoresis. This protein group includes HMGA, HMGB, and HMGN, among which HMGB1 is the most abundant. HMGB1 functions as a DNA chaperone in cells [52] and functions as a DAMP outside cells. This protein can be released through active generation by activated immune cells (e.g., macrophages or monocytes) or through passive release during cell damage/death following infection or tissue injury [53]. HMGB1 is known to be released during pyroptosis [54], ferroptosis [55], and late stages of apoptosis [56], among the different types of cell death. The excessive release of HMGB1 is critically associated with inflammation during sepsis or endotoxemia in infectious diseases and sterile injuries [53,57]. HMGB1 binds to certain PAMPs and other pro-inflammatory molecules (e.g., LPS), and this enables it to interact with diverse cell surface receptors [53]. The HMGB1 complexes with cofactors operate by binding to a wide range of receptors, including RAGE, TLR4, TLR2 [58], TLR5 [59], and TIM3 [60]. Among these receptors, RAGE, TLR4, and TLR2 have been confirmed as the specific receptors of HMGB1 [57,58,61,62]. A recent finding has shown that RAGE and TLR4 operate in a feedback interaction network, which is important in HMGB1-induced inflammation [61]. The study suggested that RAGE promotes TLR4 trafficking on the cell surface via MAPK activation, whereas TLR4 increases RAGE expression. The downstream signals of RAGE and TLR2/TLR4 induce pro-inflammatory cytokine production through the NF-κB pathway and in a MyD88-dependent manner [62].

## 3. Functional Characterization of DAMPs in Vaccine and Adjuvants

Most modern adjuvants, in clinical use or in the late development stage, such as MPLA, CpG DNA, poly (I:C), and imiquimod, exert adjuvanticity through PRRs. Certain adjuvants, such as alum, emulsion, and saponins, indirectly activate innate immune responses by causing cell death at the site of administration and draining lymph nodes to subsequently generate DAMPs. Thus far, adjuvants, including alum, oil-in-emulsion (MF59 and AS03), HP-β-CD, and saponin-based adjuvants (e.g., QS21), have been shown to involve DAMPs in their mode of action.

Generally, DAMP-inducing adjuvants promote the activation of innate immune cells [63,64]. In this process, typical adjuvant-related DAMPs, including ATP, uric acid, and DNA, trigger the accumulation of neutrophils, monocytes, macrophages, and eosinophils [65]. Antigens are delivered to the draining lymph nodes via innate immune cells or passive draining to induce adaptive immune responses. Here, we review the role of DAMPs in the action of licensed vaccines or adjuvants currently in use or in phase 3 trials.

### 3.1. Non-Adjuvanted Vaccines

Several viral vaccines have been shown to trigger DAMP release or cell death signaling in the absence of an adjuvant, implying that these viruses intrinsically exhibit a DAMP-inducing property in addition to the PAMP-inducing property. When a UV-inactivated influenza virus vaccine is injected without an adjuvant, subcapsular sinus macrophage (SSM) death can be detected by microscopy. This type of macrophage death is a necrosis-like death associated with MyD88 signaling and Toll-like receptor 7 recognition. This vaccination can also trigger the release of IL-1α, which is involved in the inflammatory pathway, suggesting the upregulation of antigen presentation and humoral response [66]. Additionally, modified vaccinia Ankara virus, a viral vector actively studied for vaccine development, has been reported to induce inflammasome activation and pyroptosis in SSMs [67]. This process subsequently promotes the robust recruitment of inflammatory cells and potentiates T cell responses.

Among commercialized animal vaccines, Purevax^®^ was developed for protection against rabies, feline leukemia, and panleukopenia in cats. Although the specific mechanism and pathway have not been identified, a histopathological study confirmed the induction of necrosis with neutrophilic and macrophage inflammatory responses within seven days of vaccine injection in cats [68].

### 3.2. Alum

Alum has been shown to exert adjuvanticity through the depot effect (i.e., the slow release of antigens), and it is now accepted as a typical DAMP-inducing adjuvant (Table 1). The injection of alum causes necrotic cell death at the injection site, inducing the release of DAMPs, such as host DNA [69] and uric acids. Alum-induced host DNA then triggers IgG1 production from B cells in an interferon response factor 3 (Irf3)-independent manner and stimulates Th2 cells and IgE responses through Irf3-dependent mechanisms [70]. In contrast to the common mechanism of action of several adjuvants that trigger TLR signaling transduction, alum salts are known to require NLRP3 inflammasome activation for the production of pro-inflammatory cytokines [71]. NLRP3 activation is well known to induce the production of active IL-1β and IL-18 through cleavage by caspase 1. In particular, the phagocytosis of alum, MSU, and silica crystals leads to lysosomal destabilization and rupture. Furthermore, lysosomal damage can activate NALP3 inflammasomes. [72]. In addition, the direct administration of alum to the airway stimulates immune responses in the lungs. Exposure to alum induces cell death in alveolar macrophages and stimulates the release of IL-1α as an alarmin [73]. Transient IL-1α release in the lungs may subsequently cause delayed and sustained adjuvant effects on the lungs by inducing the formation of inducible bronchus-associated lymphoid tissue, which is a lymphoid cluster showing local immune responses associated with asthma.

### 3.3. Oil-in-Emulsion

Squalene-based emulsion adjuvants, such as MF59 and AS03, are widely used as influenza vaccine adjuvants due to their ability to induce strong immunogenic responses in humans. Clinical studies have demonstrated that these adjuvants induce robust innate immune responses required for adaptive immune system activation. Notably, ATP released from the injected muscle is known to be a crucial factor contributing to the adjuvanticity of MF59 (Table 1) [74]. In addition, the elevation of DNA and uric acid levels was observed in the serum of Addavax (AV)-immunized mice [8]. Upon injection, squalene-based oil-in-water emulsion (SE) vaccine adjuvants stimulate an immunocompetent environment through the transient release of DAMP signals, including ATP, DNA, and uric acid. These signaling molecules bind to the receptors of antigen-presenting cells (APCs), inducing cytokine and chemokine secretion, enhanced antigen uptake, and transport to draining lymph nodes [63]. However, the mechanism of action of cell death pathways underlying immune system stimulation remains poorly understood.

Findings from recent studies have suggested that the efficient phagocytosis of SE adjuvants is correlated with the loss of lymph node-resident macrophages, which induces early regulated necrosis, such as RIPK3-mediated necroptosis and pyroptosis, followed by late apoptosis, in draining lymph nodes. In particular, the role of RIPK3, a key mediator in necroptosis, in SE-adjuvanted vaccination, is crucial for CD8^+^ T cell responses. Therefore, the importance of the kinase activity suggests that necroptotic cell death in lymph node-resident macrophages is critical for the induction of CD8^+^ T cell responses and the recruitment of innate immune cells by SE adjuvants [8]. Numerous studies have highlighted the role of necroptosis in the exposure to DAMPs and the regulation of inflammatory responses [75,76,77,78]. Indeed, MF59 and AV engender mixed macrophage deaths associated with a highly immuno-stimulatory microenvironment upon immunization, characterized by the increased release of cytokines, including IL-6, IL-12, and TNF, and elevation in the levels of DNA and uric acid, compared to that in alum-vaccinated groups [8].

### 3.4. Saponin-Based Adjuvants

Saponins are composed of hydrophilic glycosides and lipophilic triterpenes and hence, qualify as detergents. They can destroy cell membranes by forming complexes with membrane cholesterol. Among saponin-based adjuvants, QS-21 is a component of the adjuvant AS01, a licensed vaccine against varicella-zoster virus. It has also been used as an approved malaria vaccine in a limited campaign in Africa. In the draining of lymph nodes, QS-21 activates caspase-1 and MyD88 pathways in SSMs and DCs to induce IL-1b and IL-18 generation, thereby inducing enhanced antibody and T cell responses. The local release of HMGB1 has been suggested to contribute to this mechanism [79]. Matrix-M™, which comprises 40 nm nanoparticles containing saponins, cholesterol, and phospholipids, is a component of NVX-CoV2373, a Novavax COVID-19 vaccine candidate undergoing phase 3 clinical trial (Clinical trial number NCT04611802).

### 3.5. HP-β-CD

The synthetic compound HP-β-CD is also a DAMP-inducing adjuvant (Table 1). HP-β-CD can be used as a vaccine adjuvant when administered via subcutaneous [80] or intranasal routes [81]. After administration, HP-β-CD induces local cell death to release host DNA, which stimulates Th2 immune responses [82]. This adjuvant, along with PAMP adjuvants, enhances immunogenicity when administered with an influenza split vaccine [83]. In addition, the recently developed Janssen COVID-19 vaccine contains HP-β-CD as an adjuvant and was allowed for use under emergency use authorization by the US FDA.

## 4. Application of DAMPs as Vaccine Adjuvants

Recently, growing evidence has corroborated that the DAMP pathway can be strategically targeted to enhance the immunogenicity of vaccines and improve the outcomes of immunotherapy.

In the application of DAMPs as adjuvants against viral infection, HSP70 was integrated with a DNA vaccine encoding an antigen for protection against HIV [84]. In draining lymph nodes of mice vaccinated with DNA encoding HIV gag and HSP70 proteins, the levels of activated DCs were significantly increased, followed by the enhancement of CD4^+^ and CD8^+^ T cell responses. The vaccine, containing HSP70, showed protective potential against HIV infection [84]. In addition, it has been suggested that HMGB1 prevents viral infections by improving protective effect of vaccines. Co-immunization with plasmid-encoded HMGB1 (pHMGB1) can enhance the vaccine efficacy for antigen-encoding DNA. In HIV vaccine studies, HMGB1 expression promoted DC maturation with CCR7 upregulation and antigen presentation, followed by increased antibody and IFN-γ responses to HIV [85]. Furthermore, the HMGB1-encoding plasmid also demonstrated adjuvant-like effects in the DNA-based influenza vaccine approach. Similar to that observed in the HIV vaccination, vaccination with plasmids encoding influenza antigen and HMGB1 can induce DC maturation by upregulating CD83, CD86, and CCR7, and mounted stronger neutralizing antibody response antigen-specific CD4^+^ and CD8^+^ T cells. Therefore, HMGB1 can be targeted to enhance the immunogenicity of viral vaccines that provide protection against viral challenges [86].

In the context of tumor immunotherapy, DAMPs have been tested extensively as immuno-stimulators. ATP, one of the most typical intracellular metabolites and an extracellular danger signal released by damaged cells, has been suggested as an effective vaccine adjuvant. Upon the injection of Ova as a model antigen with ATP, DCs underwent effective activation with increased expression of surface markers such as CD80, CD86, and MHC II, followed by augmented levels of anti-Ova IgG [87]. Interestingly, ATP may sufficiently inhibit the growth of tumors when peptide antigens that are weakly immunogenic are used. ATP-adjuvanted vaccination could reduce the levels of regulatory T cells and myeloid-derived suppressor cells and increase CD4^+^ and CD8^+^ effector/memory T cells [88]. Furthermore, HSP70 in combination with DNA vaccines resulted in prolonged protective effect against tumors. In a tumor challenge test, mice immunized with the DNA vaccine, encoding HSP70 linked with HPV16 mE7 and the human CD33 peptide gene, remained tumor-free for approximately two months, in contrast to unimmunized mice, which developed tumors within two weeks. It could generate considerably higher IFN-γ-producing CD8^+^ T cells than that observed in the control group [89].

## 5. Application of Immunogenic Cell Death (ICD) for Effective Vaccines

DAMPs can be triggered as a result of ICD pathways, and this type of cell death can also enhance vaccine efficacy through DAMP signaling pathways. ICD was initially discovered during chemotherapy and radiation therapy for cancer treatment, but it is induced in response to pathogenic infection and vaccination. ICD is a more functional term classifying various types of cell death. It indicates a form of cell death triggering not only inflammation but also adaptive immunity including antibody and T cell responses [90]. ICD has been reported to be induced during photo-based therapy as well as by oncolytic viruses and natural compounds [91]. ICD inducers can exert their anti-tumor vaccine effect by direct injection of ICD inducers or the injection of dying tumor cells (killed by the inducers) as immunogens, via DC maturation and release of immunomodulatory factors [92]. Typically, ICDs are induced via various mechanisms, such as direct ER stress (which causes ATP secretion and surface calreticulin exposure), ROS generation, and mitochondrial outer membrane permeabilization [91]. DAMPs released as a result of these reactions activate inflammatory pathways and promote phagocytosis [93].

Although it is easy to induce ICD using particular therapeutic compounds, not all chemotherapeutic agents can induce ICD. In fact, ICD-triggering agents are limited to doxorubicin, mitoxantrone, colchicine, and shikonin (although most chemotherapeutic agents cause immune suppression). These are known as “type I ICD inducers” [94]. Doxorubicin and mitoxantrone are categorized as anthracyclines and can induce ATP secretion and CD8^+^ T cell responses. Colchicine, a microtubule inhibitor, triggers the release of representative DAMP molecules, such as HSP70 and HMGB1. Similarly, shikonin induces HSP70 release and calreticulin exposure [95]. As a prototype cancer vaccine, tumor cell lysate treated with a type I ICD inducer can be utilized in order to achieve DC-mediated T cell proliferation and tumor suppression [96]. In case of cancer vaccination via photo-based therapy, such as photodynamic and photothermal therapy (“type II ICD inducers”), it has been shown that ER stress, and subsequent calreticulin exposure and enhanced DC maturation, was linked to the increase of CD8^+^ T cell responses using CT-26 and B16 tumor mouse models [97]. Moreover, CD8^+^ T cells were efficiently infiltrated into tumors and became more functional while eliciting an enhanced anti-tumor effect [97]. Oncolytic viruses, another type II ICD inducer, have been suggested as therapeutic candidates that can kill tumor cells and disrupt the immunosuppressive tumor microenvironment. Coxsackievirus, adenovirus, measles virus, Semliki Forest virus, reovirus, vaccinia virus, Newcastle disease virus, and influenza virus have been reported to display oncolytic effects. Oncolytic viral vaccines can activate stronger immune responses than other types of cancer vaccines since they harbor PAMPs and also trigger ICD-DAMP pathways [91,98]. In an experimental study utilizing vaccination of oncolytic virus-infected cancer cells, the level of ICD increased upon treatment with an oncolytic virus, and tumor growth was significantly decreased in mice [99].

During pathogenic infections, host cell receptors detect microbial patterns, such as LPS, flagellin, CpG DNA, and RNA, to induce danger signaling pathways [100]. In case of bacterial infection by *Listeria monocytogenes*, IFNα/β-induced cell death, such as STAT1-dependent apoptosis or iNOS-dependent necrosis, was induced. When host cells are infected with HIV and HCV, the expression levels of TNF-related apoptosis-inducing ligand (TRAIL), death receptor 5 (DR5), and IFNα/β from DCs are upregulated to induce T cell and B cell apoptosis [101]. Viral infection can also increase autophagy via the elF2α kinase signaling pathway related to DAMP release [102]. Although it is clear that infectious diseases are associated with ICD, the ICD pathway has not been actively targeted for the development of vaccines against infectious diseases. As new pathogens, such as SARS-CoV-2 and variants of influenza virus, continue to emerge, the development of strategies that target ICD to induce strong immunogenicity may be necessary, alongside the introduction of novel vaccine platforms.

## 6. Conclusions

**Figure 1:**
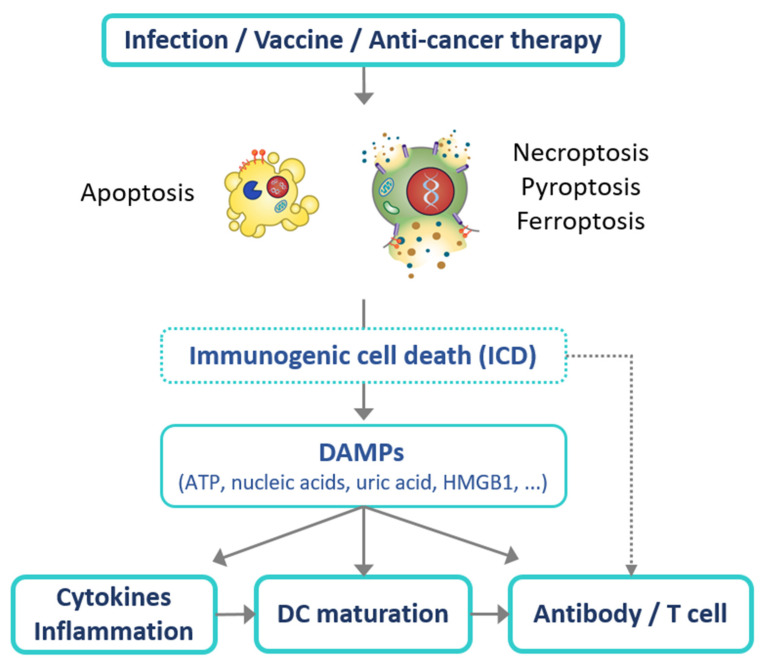
Schematic diagram depicting how DAMPs/cell death pathways affect immunogenicity during infection, vaccination, and anti-cancer therapy. Infection, vaccination, and anti-cancer therapies can induce cell death including apoptosis, necroptosis, pyroptosis, and ferroptosis. Certain types and stages of cell death are immunogenic cell death, releasing DAMPs (e.g., ATP, nucleic acid, uric acid, and HMGB1). The release of DAMPs may trigger antibody production or T cell responses by stimulating immune responses such as cytokine production, inflammation, and DC maturation.

Vaccines have advanced with technological progress, and the COVID-19 pandemic has necessitated the delivery of gene-based vaccines worldwide. Although there was a lack of comprehensive information regarding the mechanism of action of vaccines in the past, recent scientific advances have allowed us to answer this critical question. Recent discoveries have highlighted the critical roles of DAMP/cell death pathways in the induction of optimal immune response during pathogenic infections and vaccinations (Figure 1). The significance of DAMP/cell death pathways may indicate the potential for the development of the next generation of immunomodulatory agents for effective vaccines in the future.

## Figures and Tables

**Table 1:** Role of DAMPs in the action of current FDA-approved adjuvants or of adjuvants in phase 3 clinical trials.

Adjuvant	Products	DAMP	Receptor	Immune Response	Stage
**DAMP Inducer-Type Adjuvants**					
Alum salts	Numerous licensed products	DNA	TLR9	B (Ab), DC-T-cell interactions (Nat Med. 2011; 17: 996)	Approved
		Uric acid	NLR	DC (J Exp Med. 2008; 205: 869), inflammasome (J Drug Target 2018;474)	
Oil-in-emulsion	MF59	ATP	NLR	CD4 T, B (Ab) (PNAS 2013; 24: 21095)	Approved
	AS03	DNA	TLR9	B (Ab) (Sci Rep 2016; 6: 3729)	Approved
HP-β-CD	Component of Janssen COVID-19 vaccine	DNA	TLR9	Th2 (Viral Immunol 2017; 30: 463)	EUA
Saponin-based	QS21	HMGB1	RAGE, TLR2, TLR4	Ab, Th1, Th2, CD8 T (Sci Rep 2016; 6: 39475)	Phase 3
**PAMP adjuvants**					
Virosomes	Component of Inflexal V or Epaxal	Unknown	-	Ab, Th1, Th2	Approved
Liposome	MPL	Unknown	TLR4	Ab, Th1	Approved
CpG ODN	CpG 1018	Unknown	TLR9	Ab, Th1, CD8 T	Approved
**Combination adjuvants**					
Liposome, MPL, QS21	AS01	Unknown	-	Ab, Th1, CD8 T	Approved
Alum, MPL	AS04	Unknown	-	Ab, Th1	Approved

DAMP, damage-associated molecular pattern; EUA, emergency use authorization; FDA, Food and Drug Administration; DC, dendritic cell; PAMP, pathogen-associated molecular pattern.

## Data Availability

Not applicable.
